# Vascular endothelium is the basic way for stem cells to treat erectile dysfunction: a bibliometric study

**DOI:** 10.1038/s41420-023-01443-9

**Published:** 2023-05-01

**Authors:** Hede Zou, Xuesong Zhang, Wenkang Chen, Yi Tao, Bolin Li, Hanfei Liu, Ruikun Wang, Jiayou Zhao

**Affiliations:** 1 Graduate School, China Academy of Chinese Medical Sciences, Beijing, China; 2 Guang’anmen Hospital, China Academy of Chinese Medical Sciences, Beijing, China; 3 Tianjin University of Traditional Chinese Medicine, Tianjin, China; 4 Hebei University of Chinese Medicine, Shijiazhuang, Hebei China

**Keywords:** Inflammation, Regeneration

## Abstract

Vascular endothelial is considered to be a key factor in the pathogenesis of erectile dysfunction (ED). The purpose is to reveal the research trend of the field of ED and vascular endothelium. In addition, the goal is to discover the role and mechanism of vascular endothelium in ED. Bibliometrics and visualization methods based on CiteSpace were selected. We conducted the co-authorship analysis of countries, institutions and authors, co-occurrence analysis of keywords, and co-citation analysis of literature and authors through CiteSpace 6.1.R3. 1431 articles from Web of Science Core Collection (WOSCC) were included in the analysis from 1991 to 2022. We found some influential and cutting-edge nodes in each map, including countries, institutions, authors, articles, etc. Stem cell, therapy, oxidative stress, cavernous nerve injury, radical prostatectomy, fibrosis, erectile function, mesenchymal stem cell, and apoptosis may be hot keywords. In conclusion, the efficacy and mechanisms of stem cells and their derivatives in the treatment of diabetes (DM) ED and cavernous nerve injury (CNI) ED are the future research trends. Stem cells therapy for ED is a hot spot in this field, which side notes that stem cells may work mainly through improving endothelial function. Vascular endothelial cells and VEGF may repair nerve and cavernous smooth muscle directly or indirectly, and finally polish up erectile function.

## Facts

1. This study provides a research direction in the intersection of vascular endothelium and erectile dysfunction.
2. Vascular endothelium and VEGF are the basic pathway of stem cell therapy for DMED and CNI-ED.
3. Stem cell exosomes, culture medium, and gene-marked stem cells can play similar or better roles than stem cells themselves.

## Open questions

1. Whether vascular endothelium and VEGF can become a driving factor of other mechanisms and what are the deeper mechanisms in the pathogenesis and recovery of ED?
2. How necessary is the cell itself in stem cell therapy and what are the practical new methods and technologies?
3. How about the clinical efficacy and safety of stem cell therapy?

## Introduction

Erectile dysfunction (ED) is a common disease defined as failing to attain or sustain the penile erection to gain a satisfying sexual life [1, 2]. ED has obvious negative effects on patients’ health, many ED patients are depressive and anxious. Meantime, the condition has further effects on the couple’s quality of life [2]. The Massachusetts Male Aging Study indicates the prevalence of ED is about 52% in men 40 to 70 years old and the population is estimated to reach 322 million by 2025 [3, 4]. According to statistics, more than 80% of ED patients have an organic pathogen [2] and abnormal vascular endothelium emerges in the early stage of ED [5, 6]. It’s well known that ED is a predictor of cardiovascular disease (CVD), which originates from vascular endothelium dysfunction similar to ED [6]. The risk of CVD and hypertension in ED patients without vascular risk factors at first presentation exceeds 30% within 10 years [7]. It implies that endothelium dysfunction is a common etiology in the occurrence and development of both diseases. In addition, endothelium dysfunction is associated with many other diseases, such as diabetes, chronic kidney failure, tumor growth, celiac disease, etc. [8, 9].

NO/cGMP is a classic pathway of penile erection. L-arginine is transformed into NO by endothelial nitric oxide synthase (eNOS) in endothelial cells and neurotype nitric oxide synthase (nNOS) in non-adrenergic non-cholinergic (NANC) nerves. After NO enters corpus cavernous smooth muscle cells (CCSMCs) to sensitize guanylate cyclase (GC), guanosine triphosphate (GTP) is converted into cyclic guanosine monophosphate (cGMP), followed by the activation of cGMP specific protein kinase (PKG). PKG influences multiple ion channels and reduces Ca^2+^ concentration in CCSMCs, which induces the dephosphorylation of myosin. The smooth muscle of the corpus cavernous of the penis ultimately relaxes [10]. The corpora cavernous is subsequently engorged, causing the white membrane to compress the veins, preventing the outflow of blood and resulting in penile erection. Phosphodiesterase type 5 (PDE-5) is an important enzyme, inactivating cGMP and causing the rise of Ca^2+^ concentration, eventually leading to a weakening of the penis. As a result of the discovery of the pathway, phosphodiesterase type 5 inhibitors (PDE-5i) are produced and become successful drugs [11]. However, it is not always efficient following with some adverse reactions. Simultaneously, vascular endothelial dysfunction seems to be the origin of ED. Therefore, finding new therapies and potential mechanisms of vascular endothelium are beneficial and essential to remedy ED, even related conditions. In view of this, we are eager to look for the research trends of vascular endothelium in the field of ED, in order to find new effective therapies and potential mechanisms, with the ultimate goal of better treatment of ED.

CiteSpace, designed by Professor Chen Chaomei of Drexel University, is a JAVA application which is used to explore research trends in a field of knowledge by visualizing methods based on the Web of Science [12, 13]. The citation relationship of the articles can reflect the context of the development of scientific knowledge, and the sharp increase in the number of citations represents a turning point or emerging direction in the field of science, which is internal basic principle of CiteSpace [14]. It is used to spot research trends in the scientific domain here.

## Results

### The trend of publications and citations

**Fig. 1:**
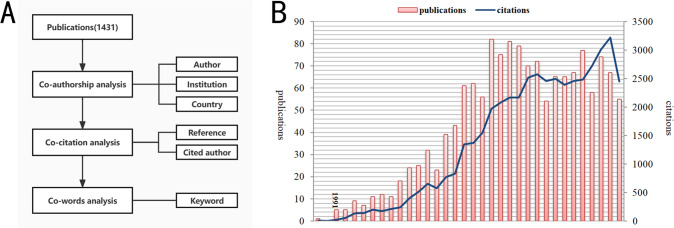
Analysis steps and basic information of literature. **A** Analysis process. **B** Trend of publications and citations.

In Fig. 1B, the number of documents and citations appears an increasing trend. The highly cited article (725 times) published in 1989, proved that diabetes damaged nerves and endothelium which regulate the relaxation of CCSMCs [15]. The largest number (82) of documents issued in 2008. The number of annual citations reaches a peak of 3221 in 2021. Since the article in 1989 has no keyword and no paper is issued in 1990, the following atlas analysis starts from 1991 to 2022.

### Co-authorship analysis

**Fig. 2:**
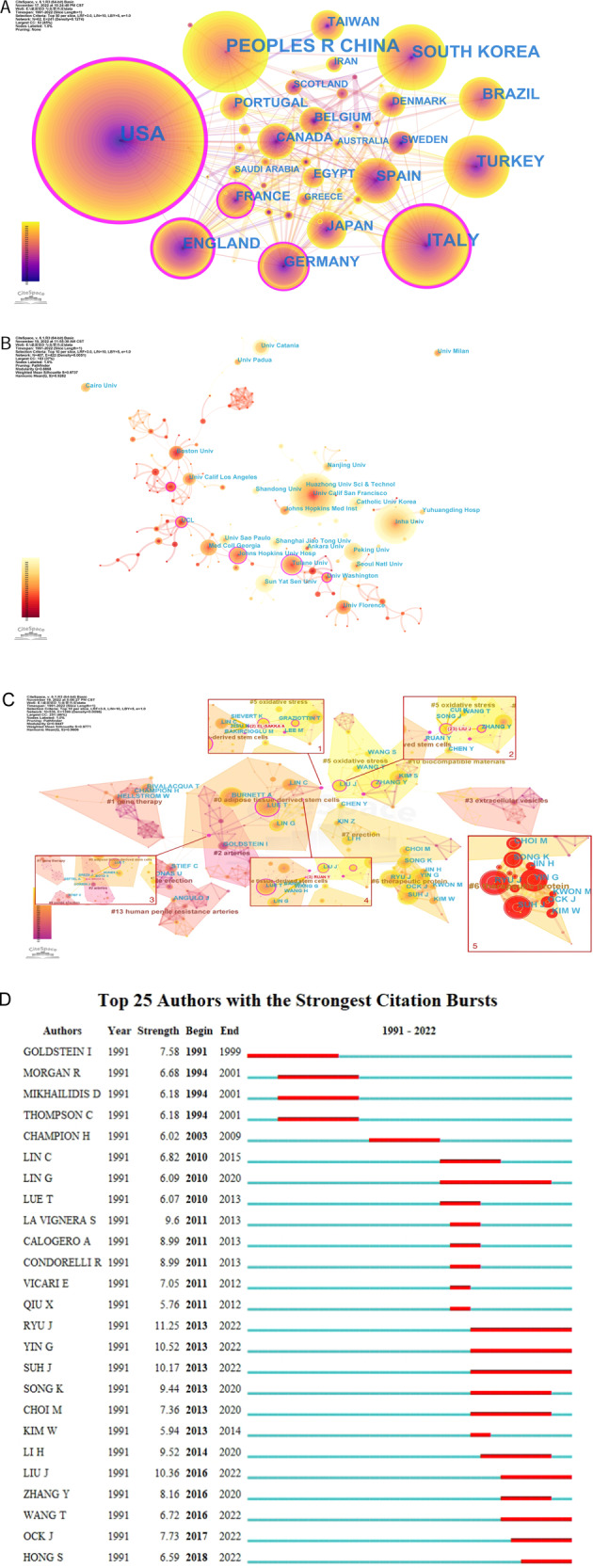
Co-authorship analyses among countries, institutions, and authors. **A** Analysis of national cooperation. **B** Analysis of institutional cooperation. **C** Cluster analysis of authors’ cooperation. **D** Bursts analysis of authors based on number of posts.

When the two co-write in an article, they form a co-authorship relationship. The United States has the largest amount of papers in Fig. 2A. Table 1 shows the top 10 countries in terms of the number of papers and the time when the first one is published. Figure 2B is the map of co-institution analysis and Table 2 shows organizations with more than 10 documents. Figure 2C is the authors’ cooperation map after clustering. It can be seen that the authors in the middle have more recent papers. In bursts analysis (Fig. 2D) of author cooperation, the red bar is the stage of a sudden increase in the number of papers, corresponding to begin time and end time, some authors have a quick growth in the number of papers published recently, such as RYU J, YIN G, SUH J, LIU J, WANG T, OCK J, HONG S, etc. Specific information can be obtained from Table 3.

**Table 1:** Top 10 countries in terms of number of documents.

Rank	Country	Year	Publications
1	USA	1991	446
2	China	2005	238
3	Italy	1996	158
4	South Korea	1997	126
5	Turkey	1995	99
6	England	1994	81
7	Brazil	2004	71
8	Germany	1991	67
9	Spain	1997	60
10	Japan	1994	44

**Table 2:** Institutions in terms of number of documents (≥10).

Rank	Institution	Year	Centrality	Publications	Country
1	Univ Calif San Francisco	1999	0.09	62	USA
2	Inha Univ	2004	0.03	52	Korea
3	Huazhong Univ Sci & Technol	2016	0.00	24	China
4	Univ Porto	2006	0.00	23	Portugal
5	Sun Yat Sen Univ	2011	0.01	23	China
6	Tulane Univ	2000	0.15	23	USA
7	Peking Univ	2010	0.09	21	China
8	Univ Catania	2011	0.01	20	Italy
9	Catholic Univ Korea	2012	0.01	18	Korea
10	Johns Hopkins Univ Hosp	2001	0.15	18	USA
11	Shanghai Jiao Tong Univ	2011	0.02	17	China
12	Boston Univ	1998	0.10	17	USA
13	Med Coll Georgia	2001	0.08	15	USA
14	Univ Roma La Sapienza	1997	0.00	15	Italy
15	Univ Sao Paulo	2009	0.00	15	Brazil
16	Univ Florence	1999	0.01	14	Italy
17	Nanjing Univ	2011	0.01	13	China
18	Seoul Natl Univ	2003	0.08	13	Korea
19	Hannover Med Sch	2000	0.00	13	Germany
20	Univ Calif Los Angeles	2002	0.02	12	USA
21	Duke Univ	2000	0.00	11	USA
22	Johns Hopkins Med Inst	2003	0.00	11	USA
23	Ankara Univ	2001	0.01	11	Turkey
24	UCL	1999	0.15	11	England
25	Cumhuriyet Univ	1999	0.00	10	Turkey

**Table 3:** Top 30 authors in terms of number of documents.

Rank	Papers	Centrality	Authors	Rank	Papers	Centrality	Authors
1	42	0.23	LUE T	16	16	0.00	LA VIGNERA S
2	40	0.03	RYU J	17	15	0.00	CONDOEELLI R
3	39	0.03	BURNETT A	18	15	0.00	CALOGERO A
4	37	0.00	SUH J	19	15	0.00	MORGAN R
5	34	0.02	YIN G	20	15	0.02	WANG T
6	29	0.04	KIM S	21	15	0.02	CHEN Y
7	27	0.06	LIN G	22	15	0.00	OCK J
8	25	0.01	LIN C	23	14	0.00	MIKHAILIDIS D
9	23	0.13	LIU J	24	14	0.00	THOMPSON C
10	23	0.01	SONG K	25	14	0.00	CHAMPION H
11	21	0.02	LI H	26	14	0.01	KIM W
12	19	0.02	BIVALACQUA T	27	14	0.05	GOLDSTEIN I
13	18	0.08	CHOI M	28	13	0.00	HONG S
14	16	0.10	STTIEF C	29	13	0.00	LEE J
15	16	0.10	ZHANG Y	30	13	0.02	HELLSTROM W

In Fig. 2A–C, some nodes have purple outer rings. Thus, it can be speculated that studies from the USA, Britain, France, Italy, Germany and some corresponding institutions, such as Tulane Univ, UCL, etc. are more influential. In Fig. 2C, four nodes with a low volume of documents but high centrality are picked out. They all connect two or more different themes. For example, El-Sakka A, collaborating with multiple authors, from Univ Calif San Francisco, manifest that intracavernous injection (ICI) of vascular endothelial growth factor (VEGF) minutes after injury is proven to improve arterial injury ED in rats and Chinese medicine mixture could elevate levels of basic fibroblast growth factor (bFGF) and caveolin-1 expression of penis tissue to antagonize adverse effects of high cholesterol on erectile function (EF) in rats [16, 17].

LIU J, from Huazhong Univ Sci & Technol of China, and collaborators proved that adipose-derived stem cells (ADSCs) could increase the secretion of insulin-like growth factor-1 (IGF-1), bFGF and VEGF in endothelial cells and CCSMCs of aged SD rats to combat oxidative stress [18]. And it may be more efficient than bone marrow mesenchymal stem cells (BMSCs) for diabetic ED rats [19]. RUAN Y, one of the collaborators, verified icariside II (ICA II) and low-intensity extracorporeal shockwave therapy (Li-ESWT) could inhibit the atrophy of CCSMCs, endothelial dysfunction, and lipidoses and redound the growth of stem/progenitor cells to improve obesity-related ED [20, 21]. Brock G et al. [22] demonstrated that vitamin E could enhance the efficiency of PDE5i in treating diabetes-related ED in rats, suggesting that the use of oxygen-free radical scavengers may ameliorate EF in diabetes.

After clustering, the bursts analysis is carried out. Cluster 6 is selected for display, owing to the number of papers issued by multiple authors increases with great speed recently. They primarily used diabetic ED models to study the pathological mechanisms and molecular pathways to seek therapeutic targets.

### Co-citation analysis of references

Co-citation analyses of references and authors are included. When two documents appear in the references of citing-document at the same time, these two constitute a co-citation relationship and the same applies to co-cited author analysis. The literature co-citation networks, the most distinctive function of CiteSpace, inform us the development contexts and trends of the field [13].

**Fig. 3:**
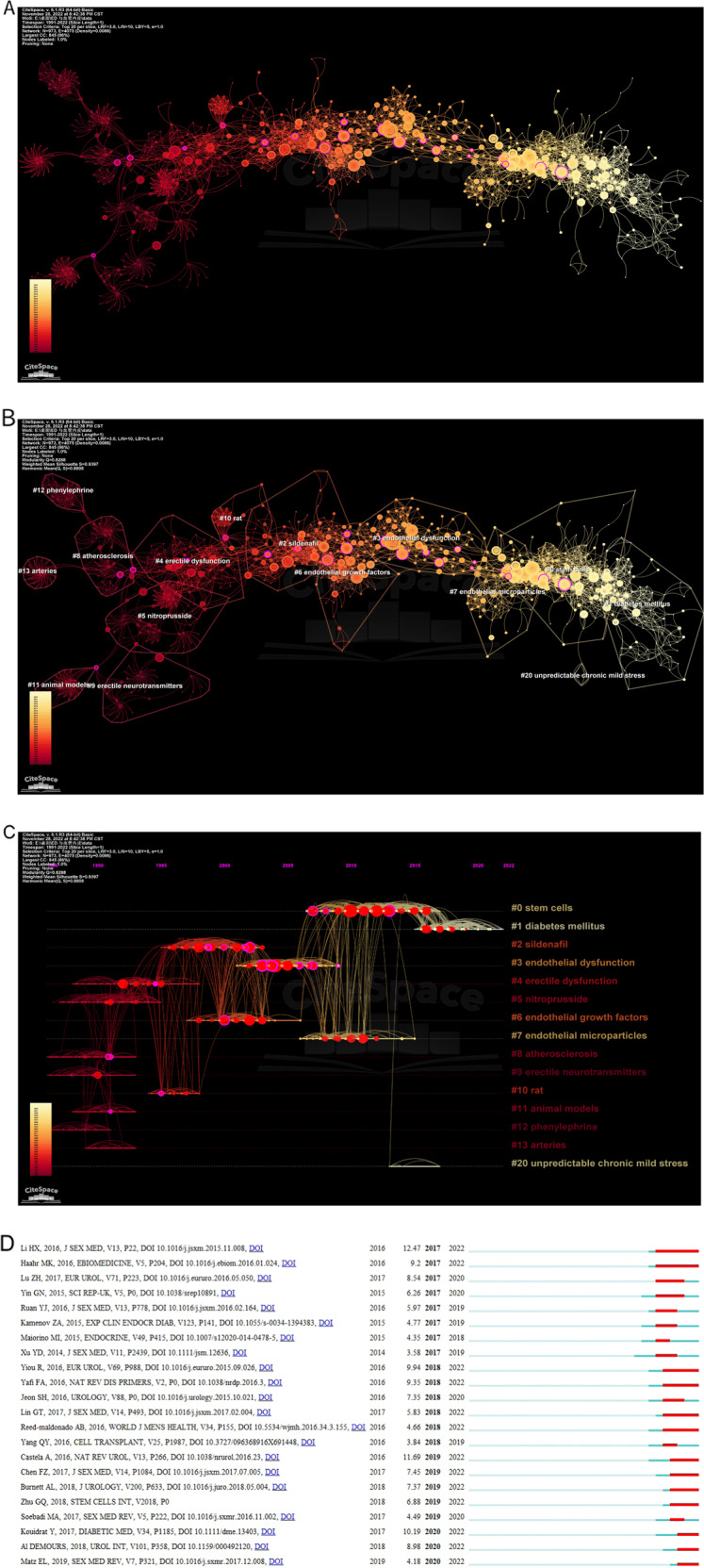
Co-citation analysis of references. **A** Co-citation of references. **B** Clusters of co-citation of references. **C** Timeline of co-citation of references. **D** References with bursts lasting until 2022.

In Fig. 3A, the color becomes lighter from left to right, implying the representative literature on the right are cited more recently. Table 4 shows the top 10 papers in co-citation frequency. Lue TF [23] reviewed the physiology of erection, the pathology, and the medication of ED. The article (Kaiser DR, 2004, J AM COLL CARDIOL) [24] suggested that ED was a risk factor for CVD. Montorsi F et al. [25] revealed that the prevalence of ED in cardiovascular patients was 49% and ED occurred 38.8 months earlier than CVD. Meanwhile, chronic therapy of tadalafil (20 mg on alternate days for 4 weeks) could improve endothelial function in patients with increased cardiovascular risk [26].

**Table 4:** Top 10 papers in terms of co-citation frequency.

Rank	Frequency	Citations (by 2022.11.17) in WOS	Centrality	Author	Year	Source	DOI
1	39	293	0.08	Kaiser DR	2004	J AM COLL CARDIOL	10.1016/j.jacc.2003.07.042
2	32	193	0.06	Albersen M	2010	J SEX MED	10.1111/j.1743-6109.2010.01875.x
3	28	191	0.03	Rosanoa GMC	2005	EUR UROL	10.1016/j.eururo.2004.10.002
4	26	115	0.02	Fandel TM	2012	EUR UROL	10.1016/j.eururo.2011.07.061
5	26	103	0.09	Garcia MM	2010	J SEX MED	10.1111/j.1743-6109.2009.01541.x
6	25	78	0.01	Lee MC	2002	J UROLOGY	10.1016/S0022-5347(01)69141-9
7	24	72	0.11	Liu GH	2013	PLOS ONE	10.1371/journal.pone.0072790
8	24	991	0.16	Lue TF	2000	NEJM	10.1056/NEJM200006153422407
9	23	426	0.28	Montorsi F	2003	EUR UROL	10.1016/S0302-2838(03)00305-1
10	23	94	0.03	Li HX	2016	J SEX MED	10.1016/j.jsxm.2015.11.008

ICI of ADSCs could repair the injured nerve cells in the major pelvic ganglia (MPG) of rats and upgrade the erectile response [27]. Albersen M et al. [28] demonstrated ADSCs improved neurogenic ED in rats through the release of intracellular preformed substances or active secretion of certain biomolecules. Garcia MM et al. [29] and Liu GH et al. [30] hold that ADSCs improves EF in diabetic ED models through paracrine or VEGF. Lee MC et al. [16] confirmed that ICI of VEGF improved EF of rats underwent bilateral ligation of the internal iliac arteries. It upturned the expression of nNOS, reduced atrophy of CCSMCs, and promoted endothelial cells proliferation and hypertrophy. These experiments suggest a potential mechanism: stem cells secrete VEGF, thereby improving the penile tissue structure.

In the 10th article (Li HX, 2016, J SEX MED), Li-ESWT effectively improved ED in rats with pelvic neurovascular injury by recruiting endogenous progenitor cells and activating Schwann cells coinciding with angiogenesis, tissue, and nerve generation [31]. These literature mainly focus on the themes on the relationship between ED and CVD, stem cells, diabetes-related ED and Li-ESWT.

Figure 3B shows clusters of co-citation of literature. The development tendency of the knowledge domain is primarily on the right. Further plotting the timeline map (Fig. 3C), the evolution of each cluster can be observed more definitely. Cluster 0, Cluster 1, Cluster 7, and Cluster 20 may be the coming trends. Nevertheless, they are all named with keywords in maps. If the titles and abstracts of the articles are used for naming, unlike results will be obtained in Table 5. The lines between Cluster 0 and Cluster 1 and Cluster 7 and Cluster 3 are dense, it can be further deduced that stem cells repair the nerve and endothelial damage caused by diabetes to treat ED.

**Table 5:** Cluster naming under three methods.

Cluster	Keywords	Titles	Abstracts
#0	Stem cells	Cavernous nerve injury	Stell cell
#1	Diabetes mellitus	Narrative review	Regenerative therapy
#7	Endothelial microparticles	Arterial erectile dysfunction	Healthy men
#20	Unpredictable chronic mild stess	TNF-α treatment	ucm

In Table 6, we enumerate some documents from Fig. 3D, whose bursts stage last until 2022, representing hot articles recently. Here we briefly introduce these papers. Stem cell therapy is considered promising but needs more extensive research [2, 32, 33]. In the article (Haahr MK, 2016, EBIOMEDICINE) [34], adipose-derived regenerative cells (ADRCs) improved ED caused by radical prostatectomy (RP) and showed good tolerance. A phase 1/2 pilot clinical trial (Yiou R, 2016, EUR UROL) [35] verified the bone marrow-mononuclear cells (BM-MNCs) had similar healing effects. Another open-label phase 1 clinical trial (Al DEMOURS, 2018, Urol Int) [36] gain a similar efficiency of bone marrow-derived mesenchymal stem cells (BM-MSCs) with good safety and tolerance.

**Table 6:** References with bursts of citations lasting until 2022.

Frequency	Citations (by 2022.11.17) in WOS	Year of publication	First author	Bursts strength	DOI	Cluster
17	94	2016	Li HX	9.20	10.1016/j.jsxm.2015.11.008	0
16	93	2016	Haahr MK	9.35	10.1016/j.ebiom.2016.01.024	0
8	68	2016	Yiou R	4.66	10.1016/j.eururo.2015.09.026	0
19	277	2016	Yafi FA	11.69	10.1038/nrdp.2016.3	0
23	51	2016	Lin GT	12.47	10.1016/j.jsxm.2017.02.004	1
17	28	2016	Reed-maldonado AB	9.94	10.5534/wjmh.2016.34.3.155	1
10	72	2017	Castela A	5.83	10.1038/nrurol.2016.23	1
13	57	2017	Chen FZ	7.45	10.1016/j.jsxm.2017.07.005	1
12	278	2018	Burnett AL	7.37	10.1016/j.juro.2018.05.004	1
12	41	2018	Zhu GQ	6.88	10.1155/20181/1302672	1
17	147	2017	Kouidrat Y	10.19	10.1111/dme.13403	1
15	44	2018	Al DEMOURS	8.98	10.1159/000492120	1
7	33	2019	Matz EL	4.18	10.1016/j.sxmr.2017.12.008	1

The incidence rate of ED in diabetes patients is about 52.5%, roughly 3.5 times that of healthy people [37]. The pathogenesis of diabetic ED is complex, but the disorder of endothelial function is the core [38]. After injection of endothelium-independent vasodilators, penis erection can be achieved in diabetes patients [15]. In the article (Chen FZ, 2017, J SEX MED) [39], ADSC-derived exosomes (ADSC-Exo) had equal efficiency to ADSCs in treating diabetic ED in rats, compared to the control group treated with phosphate-buffered saline (PBS). Particularly, they observed an increase in endothelial markers and a decrease in endothelial apoptosis. A combination of ADSCs and Li-ESWT could better recover diabetic ED in rats than a single method [40]. Lin GT et al. [41] suggested one of the mechanisms of Li-ESWT as a non-invasive treatment is to activate the endothelial progenitor cells of the penis.

The last article (Burnett AL, 2018, J UROL) [42] provides a guideline for diagnosing and treating ED.

### Co-citation analysis of authors

**Fig. 4:**
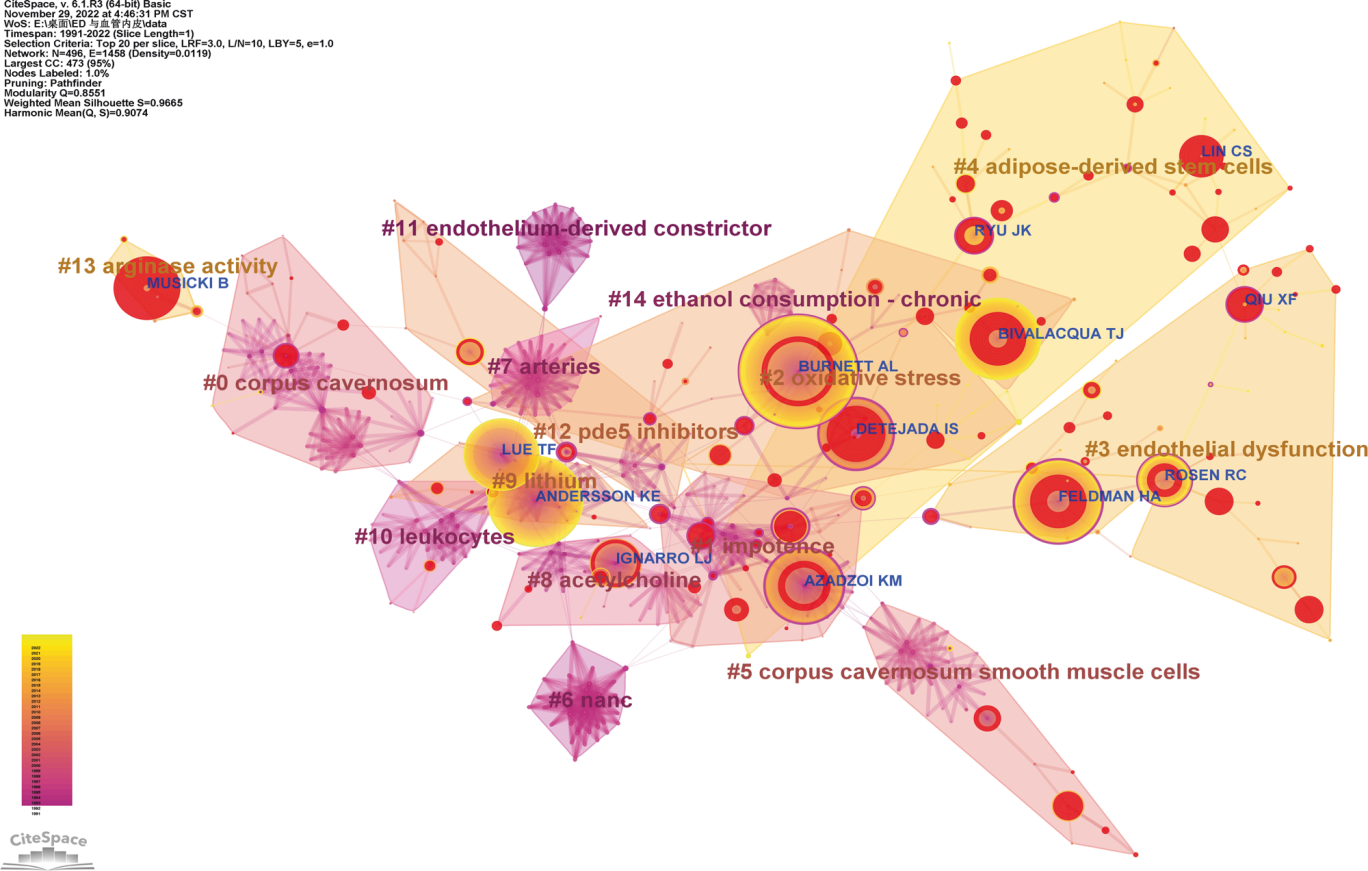
Co-citation analysis of authors.

If an author is cited more often, he can be considered influential. If the number of recent citations increases rapidly, it implies his research may be a hot topic. Some authors having high influence are revealed in Fig. 4 and some of them are shown in navy blue words. Cluster 2–4 and 13 may represent the tide of research, involving some typical researchers, who have higher and more recent citations. Table 7 shows the authors with citations more than 100 and their clusters (named by keywords). After extracting the cluster names from the title, the four mentioned are #2 endothelial nitric oxide synthase, #3 endothelial microparticle, #4 cavernous nerve injury, and #13 potential role, respectively.

**Table 7:** Authors with co-citation frequency ≥100.

Rank	Frequency	Centrality	Cited-authors	Cluster
1	378	0.15	BURNETT AL	2
2	283	0.03	BIVALACQUA TJ	4
3	264	0.04	ANDERSSON KE	9
4	262	0.25	FELDMAN HA	3
5	214	0.23	AZADZOI KM	1
6	205	0.02	MUSICKI B	13
7	205	0.23	DETEJADA IS	2
8	194	0.04	LUE TF	9
9	153	0.19	ROSEN RC	3
10	133	0.10	IGNARRO LJ	8
11	119	0.02	LIN CS	4
12	110	0.11	QIU XF	3
13	101	0.18	RYU JK	4

### Co-words analysis of Keywords

**Fig. 5:**
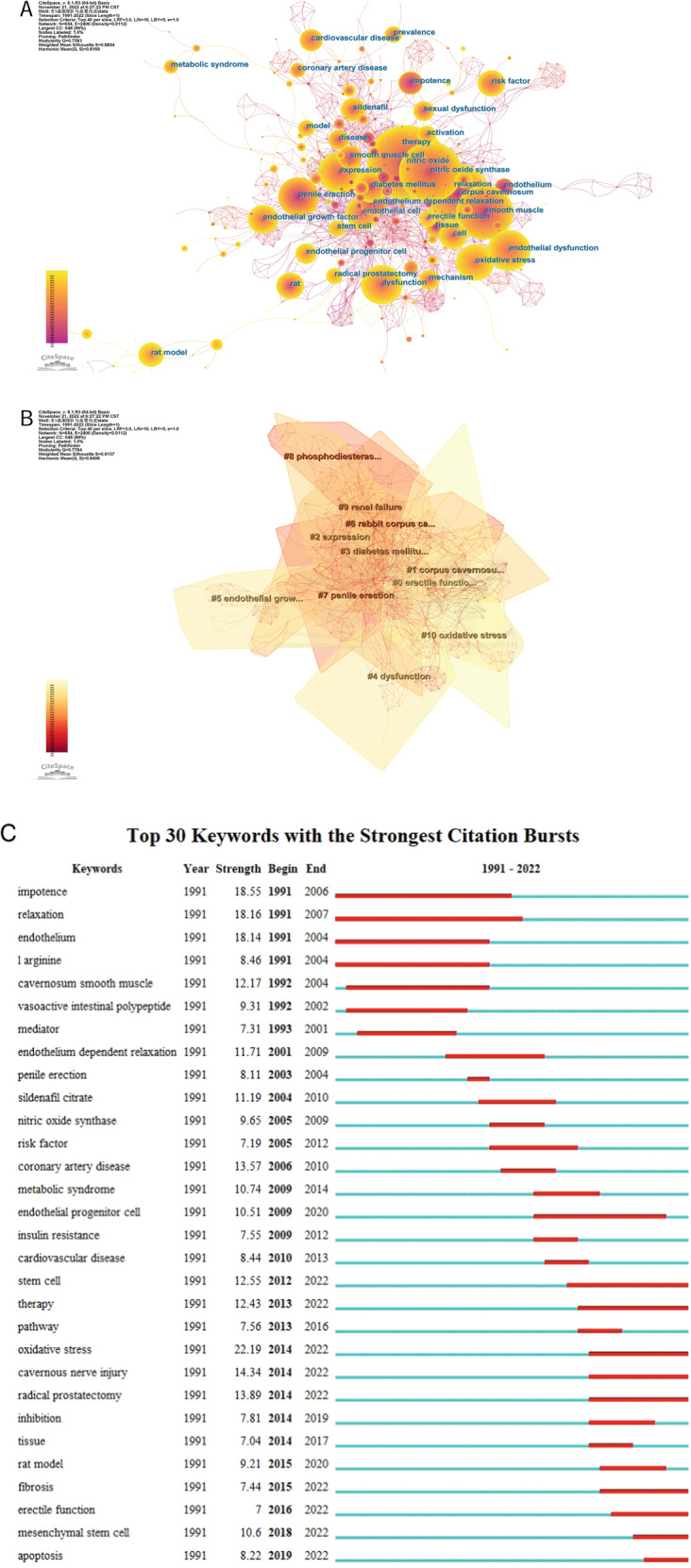
Co-words analysis of keywords. **A** Co-occurrence of keywords. **B** Clusters of keywords. **C** Bursts analysis of keywords.

The co-occurrence analysis of keywords help us learn the development trends and hotspots in the research field. The keywords with a frequency more than 50 are displayed in Fig. 5A and Table 8. But the keywords erectile dysfunction and men are not exhibited because of the demand for clear maps. Figure 5B shows clusters with more than 30 keywords, which have 11 research topics. Cluster 0, Cluster 2–5, and Cluster 10 have lighter colors, suggesting that there may be many recent studies on these topics. To further seek the research trends and hotspots in the field of ED and vascular endothelium, we conducted keyword bursts analysis (Fig. 5C). The top 30 keywords with the strongest citation bursts are shown. It implies that stem cell, therapy, oxidative stress, cavernous nerve injury, radical prostatectomy, fibrosis, erectile function, mesenchymal stem cell, and apoptosis may be hot spot issues.

**Table 8:** Keywords with frequency ≥50.

Rank	Frequency	Centrality	Keywords	Rank	Frequency	Centrality	Keywords
1	885	0.02	Erectile dysfunction	21	83	0.20	Endothelium-dependent relaxation
2	426	0.01	Nitric oxide	22	82	0.05	Impotence
3	313	0.06	Nitric oxide synthase	23	82	0.04	Sildenafil
4	306	0.01	Corpus cavernosum	24	79	0.03	Cardiovascular disease
5	251	0.01	Smooth muscle	25	77	0.00	Endothelial progenitor cell
6	195	0.00	Endothelial dysfunction	26	75	0.03	Smooth muscle cell
7	180	0.00	Dysfunction	27	67	0.02	Sexual dysfunction
8	177	0.01	Men	28	67	0.03	Disease
9	176	0.01	Expression	29	62	0.02	Mechanism
10	166	0.03	Oxidative stress	30	61	0.04	Coronary artery disease
11	149	0.01	Penile erection	31	61	0.01	Activation
12	123	0.02	Diabetes mellitus	32	60	0.00	Model
13	111	0.06	Endothelial cell	33	59	0.10	Tissue
14	109	0.09	Endothelial growth factor	34	58	0.09	Radical prostatectomy
15	102	0.01	Risk factor	35	57	0.07	Endothelium
16	98	0.01	Relaxation	36	57	0.01	Stem cell
17	97	0.06	Rat model	37	53	0.05	Erectile function
18	90	0.00	Cell	38	52	0.01	Metabolic syndrome
19	87	0.02	Therapy	39	51	0.01	Prevalence
20	84	0.01	Rat				

## Discussion

From the results of literature analysis, we can attain certain meaningful messages. In the co-authorship analysis and co-citation analysis of authors, influential and active researchers emerge plainly, helping strengthen communication and cooperation and ultimately promote our own development. This is the significance of these two analyses.

Keywords co-occurrence and literature co-citation analysis provide us with research trends and hotspots. The clinical and animal research of stem cells, diabetes mellitus (DM) ED, and cavernous nerves injury (CNI) ED probably be the future trends. Under this retrieval mode, these hot spots were found, indicating all three were closed related to vascular endothelium.

### Vascular endothelial dysfunction and VEGF reduction promote the development of DMED and CNI-ED

Endothelial dysfunction is supposed to be the main pathological factor of diabetes-related ED [38]. Hyperglycemia and oxidative stress in diabetes lead to vascular damage and reduce the blood supply of the corpus cavernous. Concurrently, hyperglycemia and advanced glycation end products promote oxidative stress and the generation of superoxide free radicals, activating multiple pathways and ultimately impelling endothelial cell injury and apoptosis [30, 40, 43]. These factors lead to a reduction in eNOS and VEGF synthesis, while eNOS is involved in NO/cGMP pathway and VEGF is a vital nutritional factor, which promotes angiogenesis and endothelial proliferation, increasing penis blood inflow [40]. Furthermore, the differentiation and regulation function of endothelial progenitor cells of diabetic patients is inhibited, injuring vascular endothelial function [38].

ED is common after RP, caused directly by nerve injury [44]. But endothelial dysfunction is also important in the development of CNI-ED. An experiment of CNI-ED model showed that endothelial repair was the main reason for EF recovery [45]. Whether for DMED or CNI-ED, many experiments demonstrated the broad and important role of VEGF, which promoted neural repair and increase the expression of nNOS [40] and showed neuroprosthetic effect in vitro as well [46]. Besides, VEGF activated the PI3K/AKT/mTOR pathway, stimulating autophagy to combat apoptosis of tissues in the corpus cavernous [40]. Additionally, VEGF can promote angiogenesis, the increase of blood inflow bringing more oxygen and nutrients to repair nerves and CCSMCs [29, 40]. It is thus discernible that vascular endothelium and VEGF can promote the restoration of EF.

### Mechanism of stem cell therapy for DMED

Animal experiments, listed in Table 9, identify the efficacy of stem cells and explore the mechanisms. ADSCs and MSCs are the most common types, in addition to USCs [43], UVECs, and AFSCs [47]. Almost all testified various stem cells could improve the ICP/MAP ratio in rats by diversified mechanisms. But Galhom RA et al. [43] verified the efficacy through the apomorphine. Recently, the efficacy of stem cells is considered to be exerted through secretion rather than differentiation. Therefore, many researchers have conducted experiments with exosomes, gene transfer, and conditioned medium (CM) and seem to have verified this viewpoint.

**Table 9:** Animal studies of stem cell therapy for ED.

Year	First author	Cause	Age	Intervention and counts	EF of SCs	Tests
2022	Feng H [51]	DM	7-week-old 24 SD rats and 25 ZDF rats	Control (PBS) 6 SD + 7ZDF DM 5 SD + 5ZDF DM + ICI of HUCMSCs 7 SD + 7ZDF DM + VI of HUCMSCs 6 SD + 6ZDF	ICPmax/MAP ratio ↑	smooth muscle/collagen ratio ↑ iron content and MDA ↓ SOD ↑ endothelial content ↑ eNOS, nNOS expression ↑
2022	Galhom RA [43]	DM	Young adult Wistar rats	Control 10 DM + PBS 8 DM + USCs 8 DM + USC-L 8	signs of erection in USCs (87.5%) and USC-L (93.7%)	collagen/smooth muscle ratio ↓ α-SMA and Desmin ↑
2019	Ouyang B [50]	DM	24 SD rats	Control 8 DM + PBS 8 DM + hUSC-EVs 8	ICP ↑ ICP/MAP ratio ↑	endothelial content ↑ eNOS, P-eNOS, nNOS expression ↑ smooth muscle cell/collagen ratio ↑
2018	Zhu GQ [40]	DM	8-week-old SD rats	Control 10 DM + PBS 10 DM + MSCs 10 DM + Li-ESWT 10 DM + MSCs+Li-ESWT 10	ICP/MAP ratio ↑	PECAM(CD31) expression ↑ VEGF, NGF, BDNF expression ↑ PI3K/AKT/mTOR and NO/cGMP pathways ↑ SDF-1 expression ↑ LC3 expression ↓, PARP expression ↑
2017	Chen FZ [39]	DM	6-week-old SD rats	Control 8 DM + PBS 8 DM + ADSCs 8 DM + ADSC-Exo 8	ICP/MAP ratio ↑	CD31 expression ↑ α-SMA expression ↑ smooth muscle/collagen ratio ↑ Bcl-2 ↑ caspase-3 ↓
2017	Zhu LL [48]	DM	10-week-old SD rats	Control 8 DM + PBS 8 DM + ADSCs 10 DM + M-ADSCs 10	ICP/MAP ratio ↑	VEGF expression ↑ smooth muscle and endothelial contents ↑
2017	Zhou F [49]	DM	8-week-old SD rats	Control (PBS) 8 DM + PBS 8 DM + ADSCs 20 DM + ADSC-MTs 20	ICPmax ↑ ICP/MAP ratio ↑	endothelium and smooth muscle cells content ↑ VEGF, nNOS, NGF expression in DN and MPG ↑ TSG-6 ↑
2013	Liu G [30]	DM	10-week-old SD rats	Control 12 DM + PBS 12 DM + ADSCs 12 DM + VEGF 12 DM + ADSC-VEGF 12	ICP ↑ ICP/MAP ratio ↑	endothelium and smooth muscle cells content ↑ VEGF, eNOS, VEGF R1, VEGF R2 ↑ NG2, CD 146 expression ↑
2010	Garcia MM [29]	DM	23-week-old ZDF rats	DM + PBS 10 DM + ADSCs 10	ICP ↑ ICP/MAP ratio ↑	endothelial content ↑ smooth muscle/collagen ratio (-) nNOS-positive nerve fibers ↑
2022	Ti Y [46]	CNI	8-week-old SD rats	Control 12 CNI + PBS 12 CNI + ADSCs 12 CNI + CBMSCs 12	ICPmax/MAP ratio ↑	endothelial content ↑ smooth muscle/collagen ratio ↑ nNOS expression ↑ caspase-3 ↓
2022	Kim SG [52]	CNI	9-week-old SD rats	Control (sham) CNI + PBS CNI + MSC-CM 10% CNI + MSC-CM 50% CNI + MSC-CM 100%	ICP/MAP ratio ↑ (50% and 100% groups)	eNOS, vWF expression ↑ nNOS-positive content ↑ smooth muscle/collagen ratio ↑
2022	Shao J [57]	CNI	8-week-oldSD rats	CNI + PBS 5 CNI + Gel 5 CNI + Gel+EPO 5 CNI + Gel+ADSCs 5 CNI + Gel+EPO + ADSCs 5	ICPmax/MAP ↑ relative total ICP/MAP ↑	eNOS, nNOS, α‐SMA expression ↑ smooth muscle/collagen ratio ↑ GFAP expression ↓, Tuj 1 expression ↑ caspase‐3, BAX expression ↓ Bcl‐2 expression ↑
2021	Liang L [54]	CNI	6–8-week-old SD rats	Control (sham) 6 CNI + PBS 6 CNI + PELA 6 CNI + ADSC-Exo 6 CNI + ADSC-Exo+PELA 6	ICP/MAP ratio ↑	eNOS, nNOS, α-SMA expression ↑
2020	Yang W [53]	CNI	10-week-old SD rats	Control (sham) 6 CNI + PBS 6 CNI + ADSCs 6 CNI + ADSC-V 6 CNI + ADSC-G 6 CNI + ADSC-GV 6	ICP/MAP ratio ↑	endothelial content ↑ nNOS expression ↑ smooth muscle/collagen ratio ↑ HIF-1α ↓
2019	Gu X [47]	NV	8-9-week-old nude rats	Age matched control 8 NV + PBS 8 NV + AFSCs 8 NV + ADSCs 8 NV + UVECs 8	ICP/MAP ratio ↑	RECA-1, vWF, eNOS expression ↑ nNOS expression ↑ smooth muscle/collagen ratio ↑ α-SMA and Desmin expression ↑
2019	Chen Z [58]	CNI	8-week-old SD rats	Control (sham) CNI + PBS CNI + adMSCs CNI + iMSCs	ICPmax/MAP ratio ↑ total ICP/MAP ratio ↑	vWF, eNOS, nNOS expression ↑ α-SMA and Desmin expression ↑ penis tissue weight/body weight ratio ↑ caspase‐3, BAX expression ↓ Bcl‐2 expression ↑
2018	Li M [55]	CNI	12-week-old SD rats	Control (PBS) 12 CNI + PBS 12 CNI + ADSC-Exo 12 CNI + BMSC-Exo 12	ICP/MAP ratio ↑	vWF expression ↑ nNOS expression in DN and MPG ↑ nerve content in DN ↑ smooth muscle/collagen ratio ↑
2018	Ouyang X [56]	CNI	10-weeks-old SD rats	Control 8 CNI + PBS 8 CNI + MSCs 8 CNI + MSC-Exo 8	total ICP/MAP ratio ↑ ICPmax/MAP ratio ↑	nNOS expression ↑ smooth muscle/collagen ratio ↑ caspase-3 ↓
2016	Jeon SH [59]	CNI	8-weeks-old SD rats	Control 10 CNI 10 CNI + ADSCs 10 CNI + Li-ESWT 10 CNI + ADSCs+Li-ESWT 10	ICP/MAP ratio↑	VEGF, eNOS, nNOS, cGMP expression ↑ apoptosis index of smooth muscle and nerve cells ↓
2012	Fandel TM [27]	CNI	3-month-old SD rats	Sham 10 Sham+ADSCs 25 CNI + PBS 25 CNI + ADSCs (ICI) 25 CNI + ADSCs (PI) 25	ICP/MAP ratio ↑	nNOS expression ↑ smooth muscle/collagen ratio ↑
2010	Albersen M [28]	CNI	12-week-old SD rats	Sham+vehicle 8 CNI + vehicle 8 CNI + ADSCs 8 CNI + ADSC-lysate 8	ICP/MAP ratio ↑	the number of nNOS-positive nerve fibers ↑ smooth muscle/collagen ratio ↑ apoptosis index ↓

*PBS* phosphate-buffered saline, *HUCMSCs* human umbilical cord mesenchymal stem cells, *ZDF* Zucker diabetic fatty, *ICI* intracavernosum injection, *VI* tail vein injection, *ICP* intracavernous pressure, *MAP* mean arterial pressure, *MDA* malondialdehyde, *SOD* superoxide dismutase, *USCs* urine-derived stem cells, *USC-L* lysate of urine-derived stem cells, *α-SMA* α-smooth muscle actin, *P-eNOS* phospho-eNOS, *hUSC-EVs* extracellular vesicles (EVs) secreted by urine-derived stem cells, *SDF-1* stromal cell-derived factor-1, *NGF* nerve growth factor, *BDNF* brain-derived neurotrophic factor, *LC3* light chain 3, *PECAM(CD31)* platelet endothelial cell adhesion molecule, *PARP* Poly-ADP-ribose polymerase*, VEGF* vascular endothelial growth factor, *M-ADSCs* ADSCs (labeled with SPIONs) with magnetic field application, *SPIONs* superparamagnetic iron oxide nanoparticles, *ADSC-MTs* adipose-derived stem cells-based microtissues, *DN* dorsal nerve, *MPG* major pelvic ganglion, *TSG-6* tumor necrosis factor-stimulated gene-6*, ADSC-VEGF* ADSCs expressing VEGF, *EPO* erythropoietin, *Tuj 1* III β‐tubulin, *GFAP* glial fibrillary acidic protein, *MSC-CM* mesenchymal stem cell-conditioned medium, *PELA* poly (ethylene glycol)-poly(ε-caprolactone-co-lactide), *ADSC‐V* ADSC overexpressing VEGF, *ADSC-G* overexpressing GDNF, *ADSC-GV* ADSC overexpressing VEGF and GDNF, *NV* dual neurovascular injury, bilateral cavernous nerve + internal pudendal artery injury, *adMSCs* adipose-derived mesenchymal stem cells, *iMSCs* induced pluripotent stem cell-derived mesenchymal stem cells, *UVECs* umbilical vein endothelial cells, *AFSCs* amniotic fluid derived stem cells, *HIF-1α* hypoxia inducible factor, *RECA-1* endothelial cell antigen, *PI* perineural injection.

ADSCs raise ICP/MAP ratio by multifarious mechanisms [29, 30, 39, 48, 49]. Compared with the injured control group, the endothelial markers (CD31, vWF, eNOS), smooth muscle cells markers (α-SMA, Desmin), and nerve markers (nNOS) increase after intervention, suggesting that ADSCs restore the content of endothelium, smooth muscle cells, and nerves. Notably new methods can improve the effect of ordinary stem cells. Pericytes markers (NG2, CD 146) are elevated, indicating pericytes, a kind of stem cell, promote the regeneration of endothelial cells, simultaneously, ADSCs expressing VEGF gene shows better efficiency [30]. These indicate that endothelial repair is critical in EF recovery. Zhu LL et al. [48] guided ADSCs modified with superparametric iron oxide nanoparticles (SPIONs) with a magnetic field after injection. They found that its efficacy was better than that of simple injection of ADSCs, which may be related to the longer retention after modified. At the same time, VEGF expression is higher in M-ADSCs group than ADSCs group and ADSCs does not express vWF and α-SMA, thus precluding its differentiation into endothelial cells and smooth muscle cells. Likewise, ADSCs-based microtissues (MTs) is better than ADSCs due to a longer retention time [49]. Another reason may be the elevated TSG-6 fights penis inflammation in the MTs group. In addition, VEGF, nNOS, and NGF expression increased in DN and MPG, implying the nerve regeneration function of ADSCs and MTs [49]. Chen FZ et al. [39] found ADSCs-derived exosomes resembles ADSDs in efficacy and the changes of apoptosis-related proteins indicate that stem cells can resist apoptosis of endothelium and smooth muscle cells through a secretion way. Zhu GQ et al. [40] demonstrated Li-ESWT upturned SDF-1 and PECAM expression to recruit MSCs, elevating VEGF, enhancing its effect. USC-L and hUSC-EVs showed the same good curative effect and raised endothelium content as stem cells [43, 50]. Moreover, Feng H et al. [51] found that HUCMSCs can inhibit ferroptosis to protect cavernous tissue and raise the level of eNOS and nNOS to recover EF of rats. These studies show that stem cells play a role through cell secretion pathway, and the improvement of VEGF and vascular endothelium is very essential for the recovery of EF.

### Mechanism of stem cell therapy for CNI-ED

The mechanisms of stem cell therapy for CNI-ED are similar to the above, involving ascending content of endothelium, nerves, and smooth muscle cells. Ti Y et al. [46] found that more neurotrophic factors existed in the CM of CBMSCs in vitro, especially VEGF and NT4, which may explain why CBMSCs is more effective than ADSCs in vivo. Kim SG et al. [52] showed that CM in 50% and 100% concentrations were most effective, and found the concentrations of angiotrophic factors (VEGF, ANG) were much higher than that of nerve factors (BDNF, NGF, GDNF) in vitro. This may inform us that the importance of VEGF and vascular endothelium in recovery of EF. Moreover, the discovery of TNF-α, IL-1ra, and IL-4 in CM showed that stem cells could also counter inflammation [52]. Albersen M [28] found that ADSC-lysate (acellular) had similar efficacy to ADSCs. Yang W et al. [53] demonstrated that GM-ADSCs is more efficient than ADSCs by expressing more VEGF or GDNF and reducing HIF-α, relieving hypoxia of endothelial cells. It can be seen that the secretion of stem cells may be the hinge. The exact effect of exosomes well supports this point [54–56]. Ouyang X et al. [56] even think exosomes can substitute for stem cells.

Apart from the above-mentioned nutritional and rehabilitate functions, stem cell therapy can restrain the apoptosis of endothelium, nerves, and smooth muscle cells [52, 56–59]. In general, on the one hand, stem cell therapy can promote tissue regeneration by nourishing and repairing vascular endothelium, nerves, and smooth muscle through various nutritional factors. On the other hand, it has anti-fibrotic, anti-apoptotic, and anti-inflammatory effects. Significantly, vascular endothelium and VEGF seem to be more critical compared with other pathways, because endothelium and VEGF can not only directly improve EF, but also promote nerve and smooth muscle restore to indirectly improve EF.

### Clinical prospect of stem cell therapy for CNI-ED and DMED

The efficiency of stem cells for CNI-ED and DMED is authenticated by several clinical trials, listed in Table 10. They all indicated that it improved EF while remaining well-tolerated and safe. Two tests found that peak systolic velocity (PSV) increased significantly [35, 60], in the contrary, it was not apparent in these two [61, 62]. Yiou R et al. [35] demonstrated the %PNORT is improved, implying the recovery of vascular endothelial function. This implies that even though the impairment of vascular endothelium may not be the most important pathological change of CNI-ED, it is vital. Higher quality clinical studies are remained to evaluate the efficacy and safety of various stem cell therapies.

**Table 10:** Clinical studies of stem cell therapy for ED.

Year	First Author	Cause	age	Population	Intervention	Outcome	Tests	SAE
2021	You D [61]	DM/CNI	57.0 ± 14.3/67.8 ± 9.1	5/5	BMSCs	IIEF-5 at 1month ↑	PSV and EDV ( + )	No
2021	Al Demour S [60]	DM	25–75	22	2 consecutive WJ-MSCs	IIEF-5 and EHS ↑	PSV basal and 20-min PSV↑	No
2021	Mirzaei M [62]	DM	50–70	20	10 for MSCs, 10 for nomal saline	IIEF-5 ↑	PSV and EDV and RI ( + )	No
2017	Al Demour S [36]	DM	49–60	4	2 consecutive BM-MSCs	EF, IIEF-15, EHS ↑	No	No
2017	Yiou R [63]	CNI	59.9 ± 3.8	6	BM-MNCs	IIEF-15 ↑	No	No
2016	Yiou R [35]	CNI	45–70	3 per group	BM-MNCs in four dose: 2 × 10^7^, 2 × 10^8^, 1 × 10^9^, 2 × 10^9^	EF, IIEF-15, EHS ↑ greater in 2 × 10^9^	PSV and %PNORT↑	No
2016	Haahr MK [34]	CNI	46–69	Continent: 11 Incontinent: 6	ADRCs	8/11 EF ↑ 0/6 EF ↑	No	No
2010	Bahk JY [64]	DM	57–87	7	UCBSCs	EF ↑	No	No

*SAE* severe adverse event, *BMSCs or BM-MSCs* bone marrow-derived mesenchymal stem cells, *PSV* peak systolic velocity, *EDV* end diastolic velocity, *WJ-MSCs* Wharton’s jelly-derived mesenchymal stem cells, *RI* resistance index, *EF* erectile function, *BM-MNCs* bone marrow-mononuclear cells, *PNORT* penile nitric oxide release test, *ADRCs* adipose-derived regenerative cells, *UCBSCs* umbilical cord blood stem cells.

## Limitations

The study’s main flaw might be the uniform selection of databases used. Another issue is that some pertinent articles won’t be featured due to the language restriction of English only.

## Conclusion

According to the study, vascular endothelium is essential for restoring EF. The improvement of vascular endothelial function and the ascension of VEGF may be an important mechanism of stem cell therapy for DMED and CNI-ED, because of the improvement of both promoting the recovery of nerve and smooth muscle function. This may be the starting point for further research on stem cell therapy. Research on stem cell therapy for CNI-ED and DMED, such as the clinical efficacy and mechanism of stem cell therapy, the novel techniques to improve the efficacy of stem cells, alternative methods, and underlying mechanisms, are the future trend. It is meaningful and needs continuous exploration to make stem cell therapy truly applied to clinical practice.

## Materials and methods

The database selected is the Web of Science Core Collection (WOSCC). The search query (TS = (erectile dysfunction or impotence or impotentia or asynodia) AND TS = (vascular endothelium or vascular endothelial cell* or endothelium or endothelial cell*)) is used to gain 1484 papers, dating from 1989 to 2022. Articles and Reviews are included by CiteSpace 6.1.R3 with a total of 1431 papers, excluding the types of proceedings paper (2), editorial (15), letter (4), meeting abstract (29), correction (2), note (1).

Three main types of analysis are conducted, namely co-authorship analysis, co-words analysis, and co-citation analysis. Analysis process and contents display in Fig. 1A. But before drawing the knowledge graphs, the publications are briefly described (Fig. 1B).

Before analysis, we need to learn how to interpret maps. (1) In knowledge maps, one node represents one object and the size of nodes or words represents frequency. For example, the larger the node, the more papers published by one author in the cooperation map of authors. This rule is also applicable to co-words maps and co-citation maps, which respectively indicate the frequency of a keyword appearing and citation frequency of papers or authors. (2) The thickness of the connection between the two nodes represents the tightness of the relationship. For example, the more articles written by two authors together, the thicker the lines between them in co-authorship analysis. (3) Color changes stand for the flow of time. Normally, the lighter the color, the closer the time. (4) High centrality (>0.1), appearing as purple outer rings of nodes, means that the node is important and may play a turning role or connect two different fields. (5) Clustering aims to summarize different themes. The smaller the ordinal number, the more objects one cluster contains. For example, cluster 0 contains more keywords than cluster 1 in co-words analysis. (6) Bursts analysis tells us the rapid growth of the frequency of objects, which stands for possible hotspots and frontiers. For example, if one paper is quoted multiple times within a certain period, it will be detected by bursts analysis and a red ring will appear at this node in co-citation maps of references.

## Data Availability

The data that support the findings of this study are available from the corresponding author upon reasonable request.
